# Development of an Aspen Plus model for catalytic transesterification with different reactor arrangements and kinetic mechanisms

**DOI:** 10.1039/d5ra07145c

**Published:** 2025-12-10

**Authors:** Suryakanta De, Ashish Kumar Thokchom, Ranjit Kumar

**Affiliations:** a Department of Chemical Engineering, Shiv Nadar Institution of Eminence Delhi NCR Uttar Pradesh 201314 India ranjit.kumar@snu.edu.in +91-0120-7170100 extn: 754

## Abstract

The era of dependence on fossil fuels will come to an end in a few decades, with a rising demand for alternative energy resources like biofuels. Major challenges in the preservation of the environment and ecosystem, *i.e.*, air pollution, waste disposal, greenhouse effect, and climate change, are brought by fossil fuels only. Therefore, mankind must rebuild and upgrade its energy sector by introducing biofuels, which will not only reduce the carbon footprint but also meet the energy demands of future civilization. Biodiesel, composed of Fatty Acid Methyl Esters (FAME), is a renewable fuel and possesses almost similar fuel properties to petroleum. It is more biodegradable, less toxic, and follows an eco-friendly process of production. The most attractive option to choose for its production is the heterogeneous catalytic transesterification process. In the present study, different kinetic models are developed for the transesterification process with triolein as feed using the Langmuir–Hinshelwood–Hougen–Watson (LHHW) mechanism or power law kinetics using Aspen Plus V12.1. The process layout in Aspen Plus is built on reasonable assumptions, kinetic parameters, and optimum conditions taken from relevant literature. The optimum conversion of 96.4% is achieved in simulation with the same optimum conditions as defined in the original experimental work. Five different Aspen models have been developed with varying configurations and reaction kinetics. A comparative study of all the models reveals that Model 1, with LHHW kinetics, is more efficient than the other two models in terms of conversion efficiency, product purity, and percentage recovery.

## Abstract 2

Modelling triolein–methanol transesterification using Aspen Plus: impact of kinetics and separation schemes.

## Graphical abstract

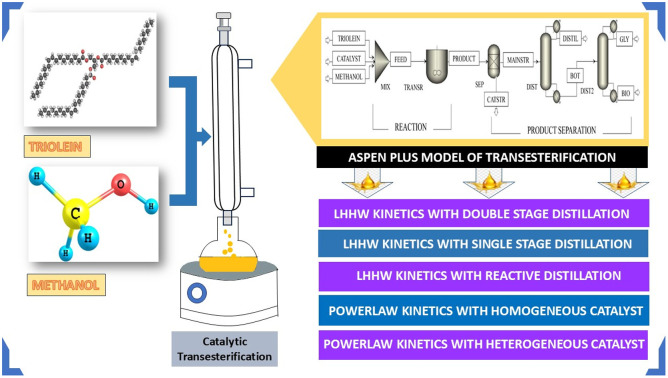

## 1 Introduction

**Fig. 1:**
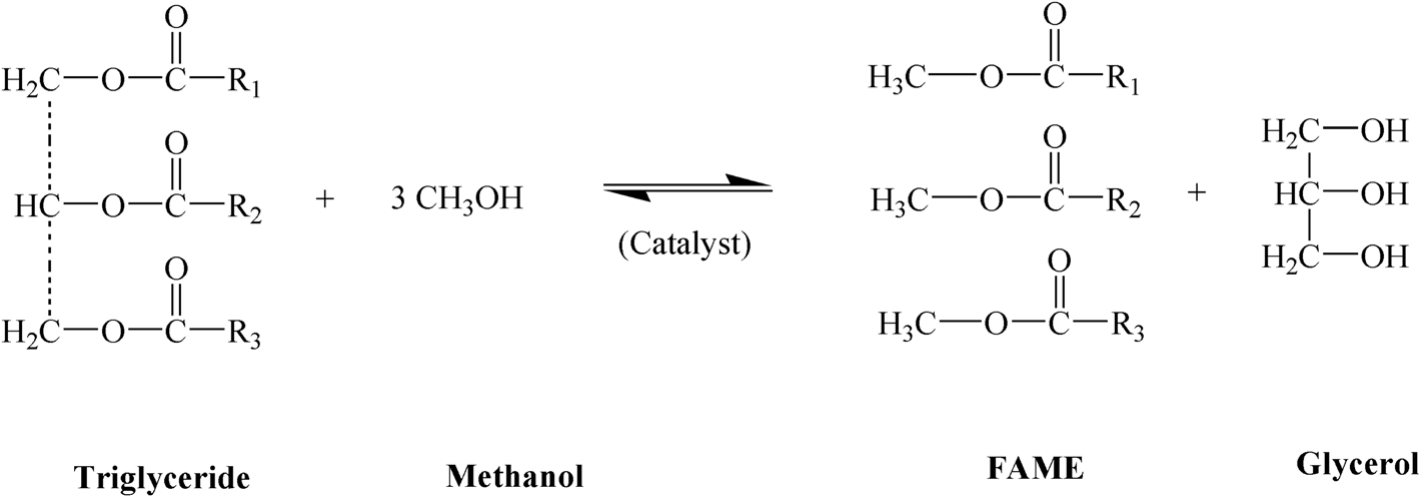
Generalized reaction of transesterification with methanol.

Dwindling oil reserves worldwide have raised concerns about an inadequate supply of liquid fuels in the market. Rather, conventional fossil fuels bring additional problems in terms of environmental pollution, greenhouse gas emissions, and climate change. This issue prompted the transportation sector across the globe to shift its reliance on renewable, green, and sustainable resources, such as biodiesel.^1–3^ Biodiesel is derived from four types of primary resources- (i) edible oils, *e.g.*, soybean oil, sunflower oil, palm oil, (ii) non-edible oils, *e.g.*, jatropha oil, mahua oil, karanja oil, (iii) waste cooking oil/animal fat, (iv) algal lipid, *e.g.*, marine and freshwater algae.^4^ The triglycerides (TG) present in these resources get converted into fatty acid methyl esters (FAME), which are basically biodiesel after reacting with some low-carbon alcohol in the presence of a catalyst. This reaction is called ‘transesterification’ as shown in Fig. 1.

The generation of FAME reduces the viscosity and improves the fuel properties over conventional diesel, such as higher cetane and octane number; combustion efficacy due to significant oxygen content (10–11%), lower sulfur and aromatic content, lower flash point, higher cloud and pour point.^5,6^ The biodiesel demand is on the rise, especially in the ‘second or third-world countries’ like Brazil, Indonesia, and India. As per the latest data provided by the International Energy Agency, the total demand for biodiesel in these nations with emerging economies will reach almost 28 billion liters per year by 2023–2028. The demand in India will reach 2.6 billion liters per year.^7^ The latest report by the World Bioenergy Association reveals that the total production of FAME biodiesel is 50 billion liters worldwide in 2023, where Indonesia contributed the highest, *i.e.*, 14 billion liters.^8^

The transesterification reaction is generally catalyzed due to the fact that the reaction conditions become severe without the use of a catalyst.^9^ Catalysis can be performed either in a homogeneous mode or in a heterogeneous mode. Moreover, the reaction can be conducted in supercritical mode by sending the reactant (alcohol) in the supercritical phase.^10^ However, heterogeneous catalysts are preferred over homogeneous ones because of ease of separation of products, no soap formation, availability of more active sites, reusability, no need for washing in the purification stage, high activity, and high quality of end products.^11–13^ Therefore, the heterogeneous catalytic transesterification is one of the major and widely accepted methods to produce sustainable green fuel, which is nothing but ‘biodiesels’ to meet the demands, quality, and standards of the transportation sector.

Although the transesterification reaction can be conducted in a laboratory with a simple experimental setup, the industrial scenario is much more complex and requires a lot of process units with complicated networks. Therefore, designing a plant-scale operation requires extensive data and complex calculations. Introducing mass and heat transfer correlations, material and energy balance, and economic assessment of the project becomes cumbersome and tedious when using manual or excel-based calculations. Simulation tools like Aspen Plus provide an efficient tool to address this challenge.^14,15^ Aspen Plus, built on FORTRAN subroutines, comprises robust computational tools for designing, optimizing, and scaling up complex industrial processes. Although there are other software options such as CHEMCAD, DWSIM, and AVEVA PRO/II, Aspen Plus remains a highly flexible and efficient tool for accurate process design in real industrial scenarios.^15–17^

Numerous researchers have conducted plant-scale to lab-scale modeling of the transesterification process for FAME biodiesel production using Aspen Plus. Silva *et al.* did a comparative study between two different pilot plants – one composed of multiple plug-flow reactors (PFR) and the other consisting of a reactive distillation column. The single reactive distillation unit shows higher ester conversion than multiple PFRs.^18^ Another comparative study was conducted by Gaurav *et al.* between two different configuration models using the Aspen Plus simulator: (i) conventional reaction–separation technology, and (ii) catalytic/reactive distillation technology. Economic assessment showed that catalytic distillation saved more capital and utility costs without compromising the conversion efficiency and product purity.^19^ Subramani *et al.* conducted the transesterification study with three different catalysts (HCl, KOH, and dolomite) and *Madhuca indica* (Mahua) seed oil as raw material. To check the economic feasibility of the process, they conducted a techno-economic study using Aspen Plus. The results proved dolomite-catalyzed transesterification to be the most economical process among the three, with the shortest payback period.^20^ Researchers have also studied the economic feasibility of the heterogeneous catalytic transesterification process and compared it with the conventional homogeneous catalytic process. The techno-economic study showed that the heterogeneous catalytic process offered a shorter payback period, a higher rate of return, and a higher net present worth than the homogeneous process.^21^ The techno-economic assessment, energy efficiency, and environmental performance of three different waste-to-biofuel production processes—hydrothermal liquefaction, transesterification, and incineration were evaluated using Aspen Plus V12. The simulation results indicated that hydrothermal liquefaction and transesterification are more environmentally friendly, with lower CO_2_ emissions, and more economically viable, offering higher net present values.^22^ In addition, Harb *et al.* prepared a kinetic model in Aspen Plus to generate biodiesel from spent coffee ground oil and achieved a biodiesel yield of 90.31% with a methanol to oil ratio of 12 : 1 and reaction temperature of 60 °C.^23^ Omoniyi *et al.* optimized the process parameters of pilot-scale biodiesel production through transesterification of waste cooking oil in Aspen Plus.^24^ With optimum operating conditions, 99.98% pure methyl oleate was produced with a yield of 109.98 kg h^−1^.

In heterogeneous catalysis, the adsorption–desorption rate is a critical factor that cannot be calculated from power law kinetics. It is an empirical equation without a mechanistic approach. On the other hand, LHHW kinetics assumes that the rate is surface-reaction controlled, *i.e.*, the first step of the reaction (triglyceride to diglyceride) is the rate-determining step. It accounts for mass transfer and adsorption–desorption steps with a mechanistic approach. Moreover, previous studies also proved that LHHW kinetics is a better option in defining the kinetics, while the power law is not adequate to explain the catalytic action [J. R. H. Ross, 2012; Ezzati *et al.*, 2020; Wu *et al.*, 2014].

**Fig. 2:**
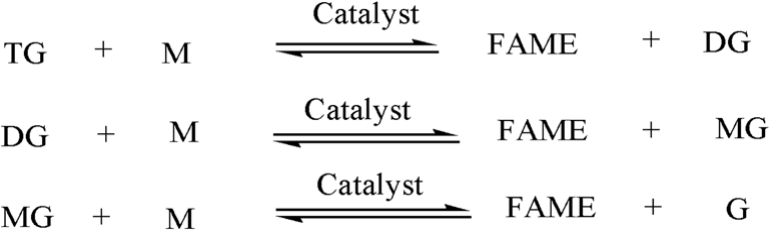
Three-step reaction mechanism of transesterification.

Although extensive research has been conducted on transesterification using Aspen Plus simulation, no model has yet been developed based on LHHW kinetics. Most studies have employed power-law kinetics or developed stoichiometric models.^25–28^ Several works have also simplified the reaction by assuming a single-step conversion of triglycerides to FAME and glycerol (G) when methanol (M) is used as the reactant.^19,29,30^ However, the actual reaction proceeds through three distinct steps, producing diglyceride (DG) and monoglyceride (MG) as intermediates. While the presence of these intermediates is undesirable, it cannot be neglected in the final product stream.^2,31,32^ The three-step reaction is illustrated in Fig. 2.

Only a few studies introduced the three-step mechanism of the transesterification reaction in their Aspen Plus model.^33–35^ Therefore, the novelty of the present study is defined as below:

i. No Aspen Plus model has been developed for the heterogeneous catalytic transesterification process with Langmuir–Hinshelwood–Hougen–Watson kinetics to date.

ii. Unlike most Aspen Plus models that assume a single-step conversion of triglycerides to biodiesel, this study adopts a more detailed and mechanistically accurate approach by implementing a three-step reaction mechanism based on Langmuir–Hinshelwood–Hougen–Watson (LHHW) kinetics. This adds uniqueness and improved predictive capability to the current work.

iii. Comparative assessment of the two kinetic mechanisms, *i.e.*, power law and LHHW, in conjunction with different reactor arrangements, and their effectiveness for the conversion to FAME biodiesel, product purity, and recovery in Aspen Plus has not been reported yet.

Therefore, the present research represents a novel contribution to the field of simulation-based catalytic transesterification using Aspen Plus. The primary objective of this study is to identify the optimal combination of kinetic mechanism and reactor configuration for the transesterification process, with the goal of maximizing conversion efficiency, FAME yield, product purity, and overall recovery.

## 2 Methodology

Five different models were developed, each with a distinct reactor arrangement and kinetics, using Aspen Plus V12.1. The description of the models is tabulated in Table 1.

**Table 1:** Models developed in Aspen Plus

Model name	Model description	Kinetic parameter reference
Model 1	Reaction separation arrangement with LHHW kinetics and double-stage distillation purification	Ezzati *et al.*^36^
Model 2	Reaction separation arrangement with LHHW kinetics and single-stage distillation purification	Ezzati *et al.*^36^
Model 3	Single reactive distillation unit with LHHW kinetics	Ezzati *et al.*^36^
Model 4	Reactor with power law kinetics for homogeneous catalytic transesterification	Salehi *et al.*^34^
Model 5	Reactor with power law kinetics for heterogeneous catalytic transesterification	Singh and Ali^37^

The kinetic equation of the LHHW mechanism followed in this study is depicted in eqn (1). This is the equation for the rate-determining step, *i.e.*, the first step of the reaction of conversion of TG to DG with methanol as reactant and 40 wt% of H_3_PW_12_O_40._*x*H_2_O/C (phosphotungstic acid to activated carbon) as heterogeneous catalyst. The other equations are developed following the same mechanism.1
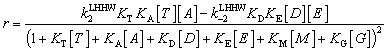


*k* ^LHHW^_2_ is the forward rate constant; *k*^LHHW^_−2_ is the backward rate constant. *K*_T_, *K*_A_, *K*_D_, *K*_E_, *K*_M_, and *K*_G_ are equilibrium constants for the adsorption of TG, alcohol, DG, FAME, MG, and G, respectively. [*T*], [*A*], [*D*], [*E*], [*M*], and [*G*] are the molar concentrations of TG, alcohol, DG, FAME, MG, and G, respectively. Models 4 and 5 utilized the power law kinetics in the form of the Arrhenius equation.

2 Reaction rate = *kT*^*n*^e^−*E*/*RT*^ × driving force

where *k* = frequency factor, *T* is the temperature of the reaction, *E* is the activation energy, *R* is the universal gas constant, and *n* is related to the interactive forces due to collision and the change in orientation of molecules with temperature, which equals zero in the case of Arrhenius equation.

### 2.1 Physical property method

In the Aspen Plus simulation, the material stream class must be defined first. The triglyceride chosen is ‘triolein’ (C_57_H_104_O_6_). The feed, *i.e.*, triolein and methanol, was defined as a conventional sub-stream. The product and by-products, *i.e.*, diolein (C_39_H_72_O_5_), monoolein (C_21_H_40_O_4_), methyl oleate (C_19_H_36_O_2_), and glycerol (C_3_H_8_O_3_), were also specified as a conventional sub-stream. Except for Model 4, the catalyst (phosphotungstic acid) is heterogeneous. Therefore, it is defined as a ‘solid’ sub-stream in the property module. The ‘Non-Random Two-Liquid model’ (NRTL) was used as a property method because it simulates the interaction between components effectively, although compounds like methanol and glycerol are considered highly polar.

### 2.2 Model assumptions

The current Aspen Plus model is based on a few reasonable assumptions to remove the complexity of the process. They are as follows-

1. Isothermal and steady-state operation of all the reactors.

2. All chemical reactions reach equilibrium.

3. The order of the transesterification reactions is assumed to be second order.^38^

4. The oil feedstock is considered to be free of any solid particles and impurities.

5. Pressure drop is neglected in all of the designed equipment.

6. No mass loss in pumps and valves, therefore not added in the design.

7. The distillation unit is an atmospheric distillation unit (ADU).

8. The triglyceride of the oil is represented by triolein.

### 2.3 Model description

Five distinct models are created to simulate the catalytic transesterification process with two different kinetics and reactor arrangements. Each model is described in the following sections.

#### 2.3.1 Model 1: reaction separation arrangement with LHHW kinetics and double-stage distillation

**Fig. 3:**
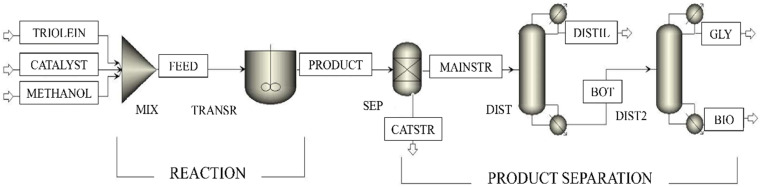
Process flowsheet of reaction separation arrangement with LHHW kinetics with double-stage distillation.

The triglyceride feed material chosen for the current study is triolein, and methanol is chosen as the reactant. The catalyst used is H_3_PW_12_O_40_.*x*H_2_O/C (phosphotungstic acid to activated carbon) as defined by Ezzati *et al.*^36^ The process flowsheet of Model 1 is illustrated in Fig. 3. Methanol is added to the ‘TRIOLEIN’ stream with a 1 : 30 triglyceride to alcohol molar ratio in a mixer (MIX). The stream ‘CATALYST’ containing the mentioned heterogeneous catalyst is also sent to the mixer. The mixed stream, *i.e.*, ‘FEED’, and fed to a Continuous stirred tank reactor (CSTR) designated as ‘TRANSR’. The transesterification reaction of triolein and methanol takes place inside the reactor. The parameters of the LHHW kinetics, *e.g.*, reaction and adsorption constants, are tabulated in Table 2. The same expression and the constants of LHHW kinetics are also used in other models. The product stream, ‘PRODUCT’, containing the methyl oleate (FAME), glycerol, diolein, monoolein, unreacted methanol, with a trace amount of unconverted triolein, is further sent to a separator named ‘SEP’, where the heterogeneous catalyst is separated. The output stream from the separator (MAINSTR) is sent to the purification unit, which contains DSTWU distillation columns in series. They are named as ‘DIST’ and ‘DIST2’ consecutively. DSTWU is a shortcut distillation unit used in Aspen Plus with less complexity, which uses the Winn–Underwood–Gilliland method. The operating conditions of the reactor and distillation columns are mentioned in Table 3. In the first distillation unit, the unreacted methanol is separated in the ‘DISTL’ stream. The bottom stream consists primarily of methyl oleate, and glycerol is purified in the second unit. There is a small amount of unconverted triolein, diolein, and monoolein, which are subsequently removed in the second distillation unit, ‘DIST2’. Glycerol, having a higher relative volatility and lower boiling point compared to methyl oleate, is separated as a distillate in the ‘GLY’.^39^ The product stream ‘BIO’, which mainly contains the biodiesel (methyl oleate), is drawn at the bottom of ‘DIST2’. The description of the Aspen modules and streams is depicted in Table 4. It is worth mentioning that all the models, including Model 1, are first simulated with the optimum conditions as defined by Ezzati *et al.*,^36^ and the conversion, yield, product purity, and recovery are determined for comparison of product quality and quantity.

**Table 2:** Kinetic parameters of LHHW kinetics

Constants	Significance	Value
k^LHHW^_2_	Forward rate constant of the rate-controlling step	4.59 × 10^−4^
K^LHHW^_−2_	Backward rate constant of the rate-controlling step	2.75 × 10^−3^
*K*_T_	Equilibrium constants for the adsorption of triglyceride	5.19 × 10^−1^
*K*_A_	Equilibrium constants for the adsorption of alcohol	1.07 × 10^−1^
*K*_D_	Equilibrium constants for the adsorption of diglyceride	1.58
*K*_E_	Equilibrium constants for the adsorption of FAME	3.7
*K*_M_	Equilibrium constants for the adsorption of monoglyceride	8.03 × 10^−2^
*K*_G_	Equilibrium constants for the adsorption of glycerol	1.58 × 10^−1^

Operating conditions for the simulation of the transesterification process in all models

**Table:** 

Feed and catalyst input	
Flow rate of triolein (mol h^−1^)	10
Flow rate of methanol (mol h^−1^)	300

**Table:** 

Parameters of reactor	
Temperature (^o^C)	65
Pressure (bar)	1
Residence time (min)	240
Catalyst loading	664
Bed voidage	0.4

**Table:** 

Parameters of the first distillation unit	
Pressure of condenser and reboiler (bar)	1
Reflux ratio	1.5 *R*_min_

**Table:** 

Parameters of the second distillation unit	
Pressure of condenser and reboiler (bar)	1
Reflux ratio	1.5 *R*_min_

**Table 4:** Description of the Aspen Plus blocks and streams in Model 1

Blocks/streams	Block/stream ID	Description and significance
Mixer	MIX	Mixes the input stream of triolein, methanol, and catalyst
R-CSTR	TRANSR	Occurrence of the transesterification reaction
Sep	SEP	Separates the catalyst from the liquid stream
DSTWU	DIST	ADU to separate the methanol
DSTWU	DIST2	ADU to separate the biodiesel (methyl oleate)
MATERIAL	TRIOLEIN	Input of triolein
MATERIAL	METHANOL	Input of methanol
MATERIAL	CATALYST	Input of catalyst (H_3_PW_12_O_40_.*x*H_2_O/C)
MATERIAL	FEED	A mixture of the input stream of triolein, methanol, and the catalyst
MATERIAL	PRODUCT	Products of the transesterification reaction with unreacted triolein and methanol
MATERIAL	CATSTR	Separated stream containing the catalyst
MATERIAL	MAINSTR	Liquid stream after catalyst separation
MATERIAL	DISTL	Distillate stream containing primarily methanol
MATERIAL	BOT	Bottom stream of unreacted triolein, diolein, monoolein, glycerol, and methyl oleate
MATERIAL	GLY	Distillate from the second distillation unit containing glycerol
MATERIAL	BIO	Bottom fraction from the second distillation unit containing biodiesel (methyl oleate)

#### 2.3.2 Model 2: reaction separation arrangement with LHHW kinetics and single-stage distillation

**Fig. 4:**
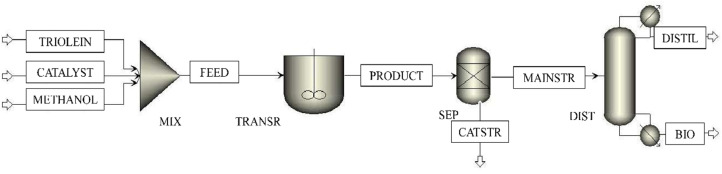
Process flowsheet of reaction separation arrangement with LHHW kinetics with single-stage distillation.

The process flowsheet of Model 2 is illustrated in Fig. 4. Methanol is added to the ‘TRIOLEIN’ stream with a 1 : 30 triglyceride to alcohol molar ratio in a mixer (MIX). The stream ‘CATALYST’, which contains the mentioned heterogeneous catalyst, is also sent to the mixer. The mixed stream, *i.e.*, ‘FEED’, is fed to a continuous stirred tank reactor (CSTR) designated as ‘TRANSR’. The transesterification reaction of triolein and methanol is occurring inside the reactor. The product stream, ‘PRODUCT’, containing the FAME (methyl oleate), glycerol, diolein, monoolein, unreacted methanol, and a trace amount of unconverted triolein, is further sent to a separator named ‘SEP’, where the heterogeneous catalyst is separated. The output stream from the separator (MAINSTR) is sent to the purification unit, which contains a single DSTWU distillation column, *i.e.*, ‘DIST’. DSTWU is a shortcut distillation unit used in Aspen Plus with less complexity, which uses the Winn–Underwood–Gilliland method. The operating conditions of the reactor and distillation columns are mentioned in Table 2. Here, the methyl oleate is extracted at the bottom (BOT), whereas methanol and glycerol with lower boiling points and higher relative volatility are separated in the upstream (DISTL).^39^ However, there are unconverted triolein, diolein, and monoolein, which can subsequently cause boiling point elevation.^40^ The description of the Aspen blocks and streams is depicted in Table 5.

**Table 5:** Description of the Aspen Plus blocks and streams in Model 2

Blocks/streams	Block/stream ID	Description and significance
Mixer	MIX	Mixes the input stream of triolein, methanol, and catalyst
R-CSTR	TRANSR	Occurrence of the transesterification reaction
Sep	SEP	Separates the catalyst from the liquid stream
DSTWU	DIST	ADU to separate the methanol
MATERIAL	TRIOLEIN	Input of triolein
MATERIAL	METHANOL	Input of methanol
MATERIAL	CATALYST	Input of catalyst (H_3_PW_12_O_40_.*x*H_2_O/C)
MATERIAL	FEED	A mixture of the input stream of triolein, methanol, and the catalyst
MATERIAL	PRODUCT	Products of the transesterification reaction with unreacted triolein and methanol
MATERIAL	CATSTR	Separated stream containing the catalyst
MATERIAL	MAINSTR	Liquid stream after catalyst separation
MATERIAL	DISTL	Distillate stream consists of unreacted methanol, triolein, diolein, monoolein, and glycerol
MATERIAL	BOT	Bottom stream of methyl oleate (biodiesel)

#### 2.3.3 Model 3: single-stage reactive distillation with LHHW kinetics

**Fig. 5:**
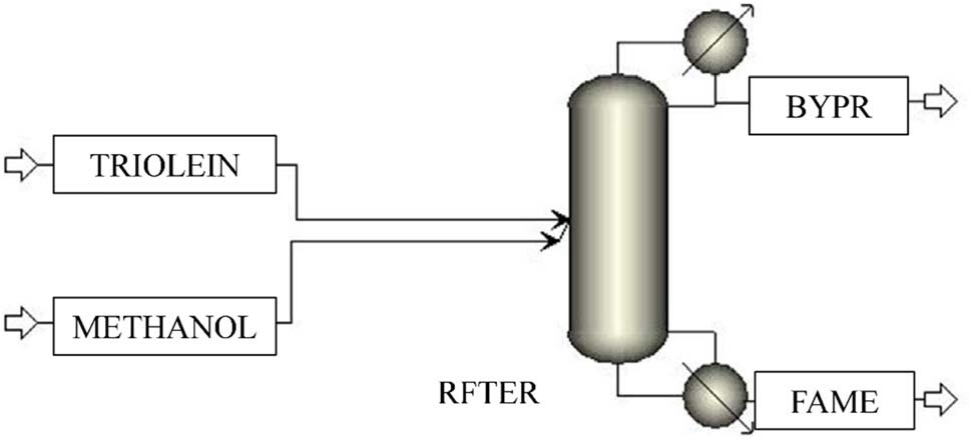
Process flowsheet of the reactive distillation unit with LHHW kinetics.

Numerous studies have been made with a reactive distillation setup for simultaneous reaction and distillation in the same unit, with the objective to eliminate the complexity of an extra separation unit and the cost associated with it, but no such research has been reported with LHHW kinetics.^19,29,40–42^ Therefore, another model, *i.e.*, Model 3, is developed in the current research work to investigate the combination of the reactive distillation unit and LHHW kinetics to meet satisfactory end-product (biodiesel) quality. ‘TRIOLEIN’, containing the triolein, and ‘METHANOL’, containing the methanol stream, enter the reactive distillation unit, which is a ‘RADFRAC’ column. Another advantage of the ‘RADFRAC’ column is that it cannot only handle non-ideal components but also handle narrow and wide boiling systems.^14,15^ The reactive distillation unit is designated as ‘RFTER’. The reaction stoichiometry of the three-step reaction with LHHW kinetics is incorporated in the block ‘RFTER’. The main product of the reaction, *i.e.*, methyl oleate (biodiesel), is drawn as a bottom product from the stream ‘FAME’. The unconverted triolein, diolein, monoolein, and glycerol are also coming in the same stream, representing an inefficient separation as well as reaction. The bottom stream (BYP) contains a low amount of product, whether it's desired or undesired. The configuration is shown in Fig. 5. The description of the block and streams is also depicted in Table 6.

**Table 6:** Description of the Aspen Plus blocks and streams in Model 3

Blocks/streams	Block/stream ID	Description and significance
RADFRAC	RFTER	Occurrence of the transesterification reaction
MATERIAL	TRIOLEIN	Input of triolein
MATERIAL	METHANOL	Input of methanol
MATERIAL	BYPR	Distillate stream consists primarily of biodiesel (methyl oleate)
MATERIAL	FAME	Bottom stream of unreacted triolein, diolein, monoolein, methanol, and glycerol

#### 2.3.4 Model 4: reactor with power law kinetics for homogeneous catalytic transesterification

**Fig. 6:**
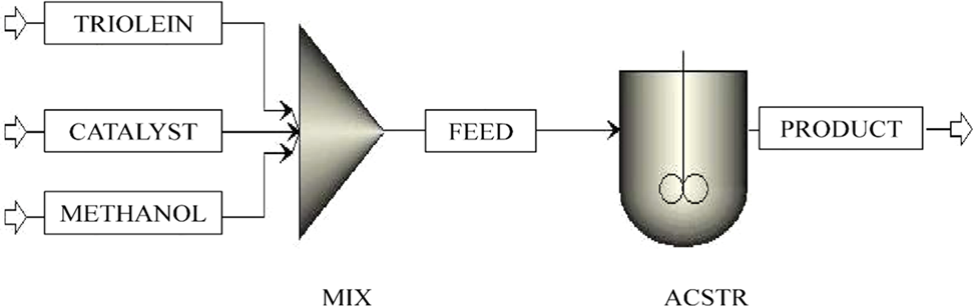
Process flowsheet of homogeneous catalytic transesterification with power law kinetics.

**Fig. 7:**
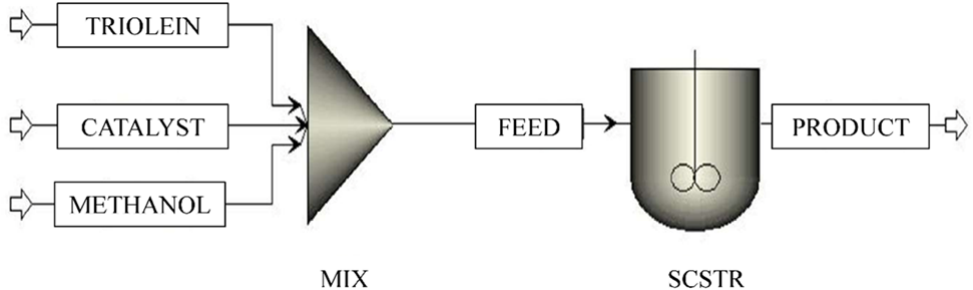
Process flowsheet of heterogeneous catalytic transesterification with power law kinetics.

Transesterification with a homogeneous catalyst is a well-practised and commonly adopted process in pilot-scale biodiesel production.^43^ In a homogeneous mode of operation, the reaction is faster, which subsequently leads to a high level of conversion. The reaction conditions are also moderate.^44,45^ Therefore, another model is built for the homogeneous catalytic transesterification with power law kinetics as defined in eqn (2). The kinetic parameters of homogeneous catalytic transesterification are taken from the study by Salehi *et al.*.^34^ Although the activation energy itself defines and introduces the catalytic effect conjugatively, the investigation of the effectiveness of power law kinetics in simulating the homogeneous catalytic transesterification process is necessary.^46^ The ‘TRIOLEIN’ stream containing triolein, the ‘CATALYST’ stream containing the homogeneous catalyst (NaOH), and the methanol stream containing the ‘METHANOL’ are first mixed in a mixer, ‘MIX’. The mixed stream, which is designated as ‘FEED’, is sent to a continuous stirred tank reactor, ‘ACSTR’. The parameters of the power law kinetics are listed in Table 7. The operating condition is the same as defined for the previous model and shown in Table 3. The ‘PRODUCT’ stream contains the main product of reaction, methyl oleate, by-products, monoolein, diolein, glycerol, and unconverted reactants triolein and methanol. No purification unit is added after the reactor. The reason is described in the following sections. The block and stream description is given in Table 8, Fig. 6 and 7).

**Table 7:** Kinetic parameters of the transesterification reaction in Model 4

Reaction	Stoichiometry	*k* (*T*_o_ = 323.15 K)	Activation energy, *E* (kcal mol^−1^)
TG → DG	C_57_H_104_O_6_ + CH_3_OH → C_39_H_72_O_5_ + C_19_H_36_O_2_	0.02311	13.5
DG → MG	C_39_H_72_O_5_ + CH_3_OH → C_21_H_40_O_4_ + C_19_H_36_O_2_	0.10659	17.4
MG → FAME	C_21_H_40_O_4_ + CH_3_OH → C_3_H_8_O_3_ + C_19_H_36_O_2_	0.05754	6.2
DG → TG	C_39_H_72_O_5_ + C_19_H_36_O_2_ → C_57_H_104_O_6_ + CH_3_OH	0.001867	10.3
MG → DG	C_21_H_40_O_4_ + C_19_H_36_O_2_ → C_39_H_72_O_5_ + CH_3_OH	0.002217	16.2
FAME → MG	C_3_H_8_O_3_ + C_19_H_36_O_2_ → C_21_H_40_O_4_ + CH_3_OH	0.000267	11.9

**Table 8:** Description of the Aspen Plus blocks and streams in Model 4

Blocks/streams	Block/stream ID	Description and significance
Mixer	MIX	Mixes the input stream of triolein, methanol, and catalyst
R-CSTR	ACSTR	Occurrence of the transesterification reaction
MATERIAL	TRIOLEIN	Input of triolein
MATERIAL	METHANOL	Input of methanol
MATERIAL	CATALYST	Input of catalyst (NaOH)
MATERIAL	FEED	A mixture of the input stream of triolein, methanol, and the catalyst fed to the reactor
MATERIAL	PRODUCT	Products of the transesterification reaction with unreacted triolein and methanol

#### 2.3.5 Model 5: reactor with power law kinetics for heterogeneous catalytic transesterification

Heterogeneous catalysts are the best alternative to overcome the difficulties introduced by homogeneous catalysts. Ease of separation of catalyst, recyclability, continuous operation, less corrosiveness, availability of more active sites, no soap formation, and the ability to convert and produce the biodiesel with a lower amount of fatty acid present in the raw material are the benefits of conducting the transesterification reaction in a heterogeneous mode.^4,47^ Therefore, another model is built for the heterogeneous catalytic transesterification with power law kinetics as defined in eqn (2). The objective of making Model 5 is the comparative assessment of two types of kinetics, *i.e.*, power law and LHHW, with the same reactant and operating conditions. In other words, Model 5 is generated to compare with Models 1 and 2. The kinetic parameters of heterogeneous catalytic transesterification are taken from the study by Singh *et al.*^37^ The ‘TRIOLEIN’ stream containing triolein, the ‘CATALYST’ stream containing the heterogeneous catalyst (potassium imbued phosphotungstic acid), and the methanol stream containing the ‘METHANOL’ are first mixed in a mixer, ‘MIX’. The mixed stream, which is designated as ‘FEED’, is sent to a continuous stirred tank reactor, ‘SCSTR’. The kinetics of transesterification reactions are incorporated in the block, ‘SCSTR’, and the parameters of the power law kinetics are listed in Table 9. The forward reactions (TG → DG; DG → MG; MG → FAME)are considered as first order and the unit of rate constant is expressed in min^−1^, whereas the reverse reactions (DG → TG; MG → DG; FAME → MG) are of second order and the unit of rate constant is in L.mmol^−1^ min^−1^. The operating condition is the same as defined for the previous model and shown in Table 3. The ‘PRODUCT’ stream contains the main product of reaction, methyl oleate, by-products, monoolein, diolein, glycerol, and unconverted reactants triolein and methanol. No purification unit is added after the reactor. The reason is described in the following sections. The block and stream description is given in Table 10.

**Table 9:** Kinetic parameters of the transesterification reaction in Model 5

Reaction	Stoichiometry	*k* (rate constant)	Activation energy, *E* (kJ mol^−1^)
TG → DG	C_57_H_104_O_6_ + CH_3_OH → C_39_H_72_O_5_ + C_19_H_36_O_2_	0.098	74.5
DG → MG	C_39_H_72_O_5_ + CH_3_OH → C_21_H_40_O_4_ + C_19_H_36_O_2_	0.651	20.3
MG → FAME	C_21_H_40_O_4_ + CH_3_OH → C_3_H_8_O_3_ + C_19_H_36_O_2_	0.876	15.7
DG → TG	C_39_H_72_O_5_ + C_19_H_36_O_2_ → C_57_H_104_O_6_ + CH_3_OH	0.009	14.5
MG → DG	C_21_H_40_O_4_ + C_19_H_36_O_2_ → C_39_H_72_O_5_ + CH_3_OH	0.008	24.1
FAME → MG	C_3_H_8_O_3_ + C_19_H_36_O_2_ → C_21_H_40_O_4_ + CH_3_OH	0.005	21.9

**Table 10:** Description of the Aspen Plus blocks and streams in Model 5

Blocks/streams	Block/stream ID	Description and significance
Mixer	MIX	Mixes the input stream of triolein, methanol, and catalyst
R-CSTR	SCSTR	Occurrence of the transesterification reaction
MATERIAL	TRIOLEIN	Input of triolein
MATERIAL	METHANOL	Input of methanol
MATERIAL	CATALYST	Input of catalyst (K^+^ impregnated H_3_PW_12_O_40_.*x*H_2_O supported over graphene oxide)
MATERIAL	FEED	A mixture of the input stream of triolein, methanol, and the catalyst fed to the reactor
MATERIAL	PRODUCT	Products of the transesterification reaction with unreacted triolein and methanol

## 3 Results and discussion

Five different Aspen models with two different reaction mechanisms (power law and LHHW), two different types of catalysts (homogeneous and heterogeneous), and with different reactor arrangements were developed. Here, we discuss the best possible combination for the transesterification process, with respect to product yield, recovery, purity, and conversion efficiency. Later, an optimization study in terms of conversion efficiency is conducted for the best-fit model, and validation is also discussed in this section.

### 3.1 Comparative analysis of conversion efficiency

**Fig. 8:**
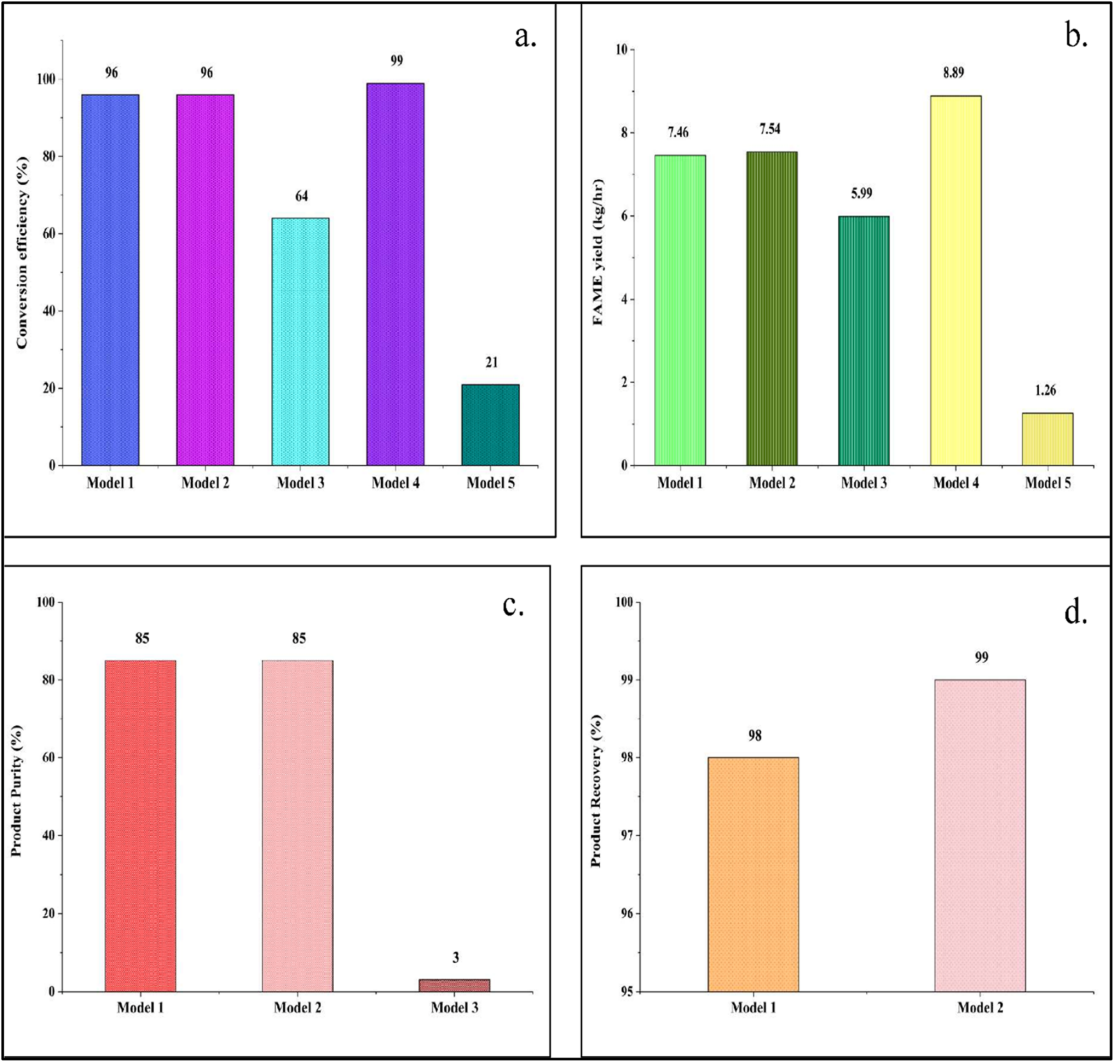
Comparative analysis of the (a) percentage conversion, (b) yield, (c) purity, and (d) recovery in the models in Aspen Plus.

The five different models (Model 1–5) simulated with the same operating conditions as mentioned in Table 3 are further compared to evaluate the best combination of reaction kinetics and catalyst for the conversion of triolein in the transesterification process. It is observed from the results that Model 4 with power law kinetics in homogeneous mode gives the best conversion of 99%. The other models have achieved a much lower conversion, except Models 1 and 2. For these two models, the conversion is 96%. Therefore, LHHW kinetics with a heterogeneous catalyst also show similar efficacy in the Aspen Plus module. It is well established that transesterification reactions catalyzed by homogeneous catalysts proceed at a faster rate compared to those involving heterogeneous catalysts, often resulting in higher overall productivity.^48,49^ The simulation results obtained in Aspen Plus reinforce this understanding, with Model 4 employing a homogeneous catalytic system achieving the highest conversion among all configurations studied. However, the power law kinetics fails to enhance the conversion efficiency of heterogeneous catalysis, with the lowest conversion of 21% only. The comparative study is shown in Fig. 8(a).

### 3.2 Comparative analysis of product yield

The yield of FAME widely varies with kinetic mechanism, reactor configuration, and phase of the catalyst.^4,10^ Simulation results in Aspen Plus show that the flow rate of FAME in the product stream is highest in Model 4. It obtains a significant value of 8.9 kg h^−1^. As discussed in the previous section, the yield of product in a reaction is directly dependent upon the conversion factor. Therefore, the best conversion obtained in Model 4, as shown in Fig. 8, supports the result of obtaining the highest yield in the same model. Similarly, the lowest yield (1.3 kg h^−1^) is obtained in Model 5 as the conversion is lowest. However, it can be observed that heterogeneous catalytic transesterification with LHHW kinetics (Models 1 and 2) can also produce a significant amount of FAME of ∼7.5 kg h^−1^, which is hardly less than Model 4. The comparison of product yield obtained in all models is shown in Fig. 8(b).

### 3.3 Comparative analysis of product purity

Acquiring a high yield or conversion efficiency doesn't define the biodiesel's quality. The produced biodiesel must meet the fuel quality and standard as defined by ASTM standards.^50^ The product stream in each model doesn't consist of methyl oleate (FAME) only. Unconverted triolein, diolein, monoolein, methanol, and by-product glycerol also come along with the biodiesel. Therefore, a subsequent requirement of the purification unit arises to avoid detrimental effects that can compromise the product quality and properties.^2,51^ If the catalyst is not separated properly, it can damage the injector pump as well as corrode engines if it remains in the biodiesel. In addition, unreacted methanol, if not separated, can damage the gaskets and seals made of natural rubber. Unconverted TG, MG, and DG can also cause turbidity.^52^ Methanol can also lead to corrosion-induced disruptions in the distillation column with liquid buildup on trays.^53^ The presence of excess free glycerol is also considered undesirable in the final product stream of biodiesel because of engine corrosion, hazardous emissions, formation of stable emulsions, and storage problems.^54^ Rather, glycerol is a valuable product and transesterification is a great route for its production.^55^ Therefore, Model 1 and 2 is constructed with a separator and distillation columns for separation and purification. Model 3 is a reactive distillation unit. Therefore, it does not require a separate distillation unit. The product purity is very low as the reaction as well as the separation didn't occur effectively with the same reaction and separation conditions as defined in Table 3. A lot of unconverted triolein and monoolein are coming along with the FAME in the product stream.

No purification unit is added in Model 4. The reactor in Model 4 is built for homogeneous catalysis with power law kinetics, where sodium hydroxide (NaOH) is used as a catalyst. Homogeneous catalysts bring complexity as well as additional cost in catalyst and product separation. To ensure stability of the transesterification reaction, the catalyst volume required is significantly high, which in turn is a precursor to the saponification reaction. Excessive soap (RCOONa) formation introduces difficulties in the separation of FAME (RCOOCH_3_) from glycerol. The saponification reaction is shown in eqn (3) when NaOH is used as a catalyst.^56–58^

3 RCOOCH_3_ + NaOH → RCOONa + CH_3_OH

In Model 5, the conversion as well as the yield of FAME is very low, as shown in Fig. 8(a) and (b). Further separation is not economically feasible. The comparative study in terms of product purity is illustrated in Fig. 8(c).

### 3.4 Comparative analysis of product recovery

It is important to calculate how much product is recovered in the final stream from the product stream from the reactor, which also defines the efficiency of separation. The percentage recovery is calculated by the following formula-4
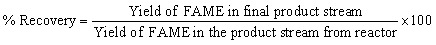


The best value (99%) of recovery is obtained from Model 2, *i.e.*, reactor-separator arrangement with a single distillation column. However, Model 1 is no less efficient in recovering the methyl oleate (FAME) in the final product stream with a recovery of 98%. Moreover, the unreacted methanol is 10 times and glycerol is 1.5 times higher in the final biodiesel stream in Model 2. These compounds are not desirable in the final extracted biodiesel, and the reason is already explained in the previous sections. In Model 3, the reaction and separation occur in the same unit. In the other two models, no separation unit is added after the reaction. Therefore, Model 1 is the most efficient in terms of product recovery. Fig. 8(d) Illustrates the comparative study of the recovery of the product obtained in Model 1 and Model 2.

### 3.5 Selection of the best model

In terms of yield and conversion efficiency, Model 4 produces the best results. The highest yield of 8.9 kg h^−1^ is obtained, and the conversion efficiency reaches 99% for the transesterification process with Model 4. Therefore, Model 4 with a homogeneous catalyst and power law kinetics gives efficacy in meeting the product standards and process efficiency. However, Model 4 can still not be regarded as the best combination, as it brings additional complexity and cost in separation, as discussed in Section 3.3. Model 3 and Model 5 do not achieve a good conversion and also fail in the proper separation of the product. Thus, Model 1 and 2 becomes the best choice in terms of conversion efficiency, product yield, product recovery, and product purity. In Model 2, the impurities, like unconverted methanol and glycerol, are higher with a single distillation unit. Therefore, it can be concluded that Model 1 with a reactor-separator arrangement with LHHW kinetics gives the best optimal result in terms of product yield, conversion efficiency, purity, and recovery. Moreover, different simulation studies involving triolein-based transesterification is compared with Model 1 in Table 11. It is noticeable that the studies either involved severe operating conditions of the production of biodiesel, or the process flowsheet is configured with more complexity, which can subsequently increase the cost. Therefore, the present model with LHHW kinetics fits best for the catalytic transesterification reaction.

**Table 11:** Comparative analysis of Model 1 with other studies

Study	Reaction temperature	Methanol to oil ratio	Catalyst weight and name	Catalyst phase	Kinetics followed	Conversion (%)
Harb & Jaoudeh (2025)	60 °C	12 : 1	1 wt%, KOH	Homogeneous	Arrhenius equation	95%
Omoniyi *et al.* (2025)	60 °C	7 : 1	CaO	Heterogeneous	Arrhenius equation	87%
Usman M. (2025)	60 °C	—	1 wt%, NaOH	Homogeneous	No kinetics followed	97%
Yadav *et al.* (2019)	95 °C	550 : 1	Nafion solid acid catalyst	Heterogeneous	Kinetic reactor not added	90%
Gaurav *et al.* (2013)	160 °C	9 : 1	3 wt%, CaO supported on Al_2_O_3_	Heterogeneous	Arrhenius equation	99.38%
This study	65 °C	30 : 1	7.5 wt%, H_3_PW_12_O_40_.*x*H_2_O/C	Heterogeneous	LHHW kinetics	96.4%

### 3.6 Optimization and validation of the best model

Model 1 was validated with the experimental work mentioned in the study by Ezzati *et al.*.^36^ The optimization study was conducted in Aspen Plus for Model 1 to find out the optimum operating parameters (methanol-to-oil ratio, reaction temperature, and catalyst weight percentage) in terms of conversion.

These three operating parameters, *i.e.*, methanol-to-oil ratio, reaction temperature, and catalyst weight percentage, are the chief controllers of the kinetics of transesterification. Reaction temperature is a critical parameter as the reaction of transesterification is endothermic in nature and requires external heating. Methanol should be added in a higher molar ratio as the reaction is reversible. This helps to shift the equilibrium towards the product (FAME) side. The reaction of transesterification is very slow without any catalyst. Therefore, the catalyst weight is an important parameter that controls conversion as well as reaction time. In Aspen Plus, sensitivity analysis was conducted to determine the percentage conversion with respect to the methanol-to-oil ratio, reaction temperature, and catalyst weight percentage for Model 1.

#### 3.6.1 Change in percentage conversion with varying methanol to oil ratio

**Fig. 9:**
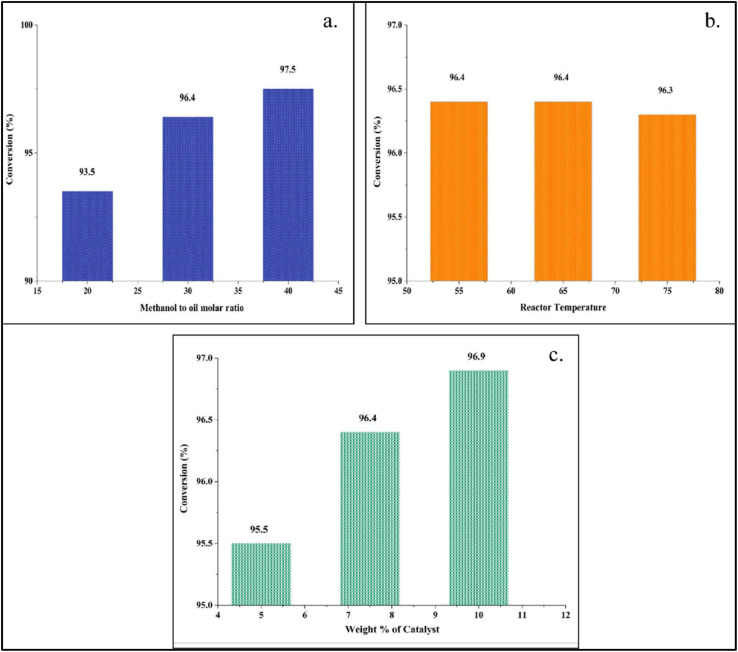
Optimization with respect to (a) methanol to oil molar ratio, (b) reactor temperature, and (c) weight percentage of the catalyst.

The transesterification reaction is reversible in nature.^59^ Therefore, a high reactant (methanol) concentration is needed to shift the equilibrium towards the product to increase the FAME yield by the forward reaction.^60^ The conversion of triolein is determined with three different methanol-to-oil ratios. At methanol-to-oil ratios (molar) of 20 : 1, 30 : 1, and 40 : 1, the conversion obtained from the results of the simulation is 93.5, 96.4, and 97.5, respectively. It is noticeable that there is no significant increase in conversion when methanol to oil ratio is increased from 30 : 1 to 40 : 1. The conversion efficiency is decreased to 93.5% when methanol to oil ratio is lower *i.e.*, 20 : 1. Therefore, the optimum conversion is determined to be 96.4% at a methanol to oil ratio of 30 : 1, which also matches with the optimum ratio as defined in experimental study. The optimization study is shown in Fig. 9(a).

#### 3.6.2 Change in percentage conversion with varying reaction temperature

Temperature is one of the critical parameters that influences the mass transfer and reaction rate in the transesterification process.^61^ The transesterification reaction is endothermic in nature. It requires external heating for the reaction to take place.^62^ In Aspen Plus, the temperature is varied at three different levels of 55 °C, 65 °C, and 75 °C to examine the conversion efficiency. The conversion efficiency is 96.4% at 55 °C and remains the same at 65 °C. The conversion efficiency decreases to 96.3% when the temperature is increased to 75 °C. This may be due to the evaporation of methanol from the reaction mixture.^62^ The optimum temperature is defined as 65 °C in the experiment. The same phenomenon can be observed in Aspen Plus and shown in Fig. 9(b).

#### 3.6.3 Change in percentage conversion with catalyst weight

The catalyst weight is also a critical parameter, as the available surface area for reaction is higher with enhanced catalyst loading, subsequently making the reaction faster.^63^ The catalyst weight percentage (catalyst to oil) is changed from 5 weight % to 10 weight %. At a weight % of 7.5, the conversion reaches 96.4% which is the same as obtained at 65 °C of temperature and 30 : 1 of methanol to oil ratio. The conversion does not change much (96.9%) when the catalyst weight% is increased to 10. Therefore, the optimum catalyst loading is determined to be 7.5 weight%. The optimum catalyst loading in the experiment is also the same. The optimization study is depicted in Fig. 9(c).

## 4 Future plan of work

The present research is a purely simulation-based work, conducted in Aspen Plus, to determine the optimal arrangement of reactor type, kinetics, and catalyst phase. However, the model is not based on any experimental investigation. Future experimental studies on transesterification using triolein-based feedstock are necessary for the successful validation of the model. In addition, the techno-economic assessment of the generated models is needed for the appropriate scale-up of the process.

## 5 Conclusion

Five different models are developed using ASPEN V12.1 to determine the best kinetic approach and best reactor configuration for FAME biodiesel production *via* transesterification. The best model is chosen with respect to four critical parameters, which determine product quality: conversion efficiency, yield, purity, and product recovery. The simulation results show that Model 1 is the best fit for the transesterification process with a conversion of 96.4% and FAME yield of 7.46 kg h^−1^. Model 1 is also validated with the operating parameters, *i.e.*, methanol to oil ratio, catalyst weight, and reaction temperature in terms of conversion efficiency as defined in the experimental study. The optimum condition from Aspen Plus simulation is found to be a methanol to oil ratio of 30 : 1, reaction temperature of 65 °C, and catalyst weight% of 7.5. The optimum conditions as determined in the simulation exactly match those of the experiment, which also proves the credibility and accuracy of the model. The present model also represents the scalability of the heterogeneous transesterification process to pilot scale. The transesterification process, as depicted in Model 1, achieves a high level of conversion (96.4%) and yield (7.5 kg h^−1^) without additional reactors, pumps, valves, or recycle streams, but simply following LHHW kinetics. It will not only subsequently reduce the production cost but will also pave the way towards sustainable, eco-friendly biofuel production.

## Conflicts of interest

The authors declare that they have no known competing financial interests or personal relationships that could have appeared to influence the work reported in this paper.

## Nomenclature

FAME: Fatty Acid Methyl Ester
LHHW: Langmuir Hinshelwood Hougen Watson
PFR: Plug Flow Reactor
TG: Triglyceride
DG: Diglyceride
MG: Monoglyceride
M: Methanol
G: Glycerol
ADU: Atmospheric Distillation Unit
*R*_min_
: Minimum Reflux Ratio

## Data Availability

The authors declare that the data supporting the findings of this study are available within the paper. Should any raw data files be needed in another format, they are available from the corresponding author upon reasonable request. The reference data used in this paper are also available online at https://doi.org/10.1016/j.renene.2020.12.055, https://doi.org/10.1002/ep.13264, https://doi.org/10.1016/j.renene.2023.02.132.
